# An *ADAMTS13* mutation that causes hereditary thrombotic thrombocytopenic purpura: a case report and literature review

**DOI:** 10.1186/s12920-021-01099-3

**Published:** 2021-10-26

**Authors:** Pengzhu Li, Jie Jiang, Qiong Xi, Zuocheng Yang

**Affiliations:** Department of Pediatrics, The Third Xiangya Hospital, Central South University, Changsha, 410013 Hunan People’s Republic of China

**Keywords:** Gene mutation, Thrombotic thrombocytopenic purpura, *ADAMTS13*, Upshaw–Schulman syndrome

## Abstract

**Background:**

Mutations in the *ADAMTS13* gene can lead to an ADAMTS13 enzyme deficiency, which is related to Upshaw–Schulman syndrome (USS). USS is a common type of thrombotic thrombocytopenic purpura (TTP). Here we present a very rare case of TTP caused by 2 mutations in the *ADAMTS13* gene. Besides, we reviewed and summarized previous pathogenic *ADAMTS13* gene mutations associated with the TTP.

**Case presentation:**

A 10-year-old female was admitted to the Third Xiangya Hospital of Central South University after experiencing discontinuous thrombocytopenia for 8 years, abnormal renal function for more than 2 years, cough for more than 10 days, and weakness of the left limb for 3 days. Gene sequencing shows the patient’s *ADAMTS13* gene contains compound heterozygous nucleotide variations: c.1335delC (p. Phe445LeufsTer52) is a frameshift variation inherited from her father and c.2130C > G (p. Cys710Trp) is a missense variation inherited from her mother. The final diagnosis was USS.

**Conclusions:**

Our study reports a very rare genetic TTP case caused by two compound heterozygous variants in the *ADAMTS13* gene. The effect of these two mutations on the secretion of ADAMTS13 requires further in vitro experiments to confirm.

**Supplementary Information:**

The online version contains supplementary material available at 10.1186/s12920-021-01099-3.

## Background

Thrombotic thrombocytopenic purpura (TTP) is a rare but critical clinical disease; its pathogenesis is mainly due to the lack of the von Willebrand factor-cleaving enzyme (ADAMTS13), and thus the von Willebrand factor (vWF) molecule is not cleaved in the plasma, and a large number of platelets aggregate and conducts widespread microvascular thrombosis. The disease is approximately 2–3 times more common in women than in men, particularly among young and middle-aged people. According to the NCBI ClinVar database, greater than 260 *ADAMTS13* mutation sites have been identified, of which 29 of 24 patients had been determined to be pathogenic mutations, as shown in Table 1. Herein, we report a unique TTP case in which new compound heterozygous nucleotide variations were confirmed by gene sequencing. To further understand the characteristics of this disease, we reviewed previously reported pathogenic cases.

**Table 1:** Pathogenic mutations in the *ADAMTS13* gene in patients with Upshaw–Schulman syndrome

Patient no	Ethnicity	Site of mutation (exon no.)	Nucleotide change	Amino acid change	Mutation type	Clinical manifestation	References
1	Turkish	exon 3	c.291_319del29	p. Glu98Profs	Frameshift	Chronic recurrent TTP	[16]
		exon 29	c.4143dupA	p. Glu1382Argfs	Frameshift		
2	Iranian	exon 23	c.2931_2936delGTGCCC	p. Cys977_Arg979delinsTrp	Frameshift	Not available	[16]
3	American	exon 17	c.2074C > T	p. Arg692Cys	Missense	Had a chronic relapsing course, responded to plasma infusion	[17]
4	American	exon 3	c.286C > G	p. His96Asp	Missense	Had a chronic relapsing course, responded to plasma infusion	[17]
5	American	exon 22	c.2851T > G	p. Cys951Gly	Missense	Had a chronic relapsing course, responded to plasma infusion	[17]
6	American	exon 13	c.1582A > G	p. Arg528Gly	Missense	Had a chronic relapsing course, responded to plasma infusion	[17]
7	American	exon 27	c.3770dupT	p. Leu1258Valfs	Frameshift	Had a chronic relapsing course, responded to plasma infusion	[17]
8	American	exon 10	c.1193G > A	p. Arg398His	Missense	Had a chronic relapsing course, responded to plasma infusion	[17]
9	American	exon 24	c.3070T > G	p. Cys1024Gly	Missense	Had a chronic relapsing course, responded to plasma infusion	[17]
10	American	exon 3	c.304C > T	p. Arg102Cys	Missense	Microangiopathic haemolysis, clinical response to plasma infusion	[17]
11	American	exon 6	c.587C > T	p. Thr196Ile	Missense	Microangiopathic haemolysis, clinical response to plasma infusion	[17]
12	American	exon 19	c.2376_2401del26	p. Ala793Profs	Frameshift	Microangiopathic haemolysis, clinical response to plasma infusion	[17]
13	American	exon 26	c.3638G > A	p. Cys1213Tyr	Missense	Microangiopathic haemolysis, clinical response to plasma infusion	[17]
14	American	_	c.1584 + 5G > A	_	Splice site	Microangiopathic haemolysis, clinical response to plasma infusion	[17]
15	Japanese	intron 3	c.331-1G > A	_	Splice site	Kidney failure and plastocytopenia	[18]
		exon 7	c.749C > T	p. Ala250Val	Missense		
16	Japanese	intron 4	c.414 + 1G > A	_	Splice site	Plastocytopenia and hemolyticanemia (the patient's parents are cousins)	[19]
17	Japanese	exon 7	c.803G > C	p. Arg268Pro	Missense	Neonatal onset and frequent relapses	[20]
		exon 12.13	c.[1342C > G;1523G > A]	p. Gln448Glu p. Cys508Tyr	Missense (haplotype)		
18	Japanese	exon 12	c.1345C > T	p. Gln449Ter	Nonsense	Neonatal onset and frequent relapses	[20]
19	American	exon 15	c.1783_1784delTT	p. Leu595Glyfs	Frameshift	Plastocytopenia and microvascular hemolysis	[21]
20	Haitian	exon 16	c.1787C > T	p. Ala596Val	Missense	Neonatal hemolysis and thrombocytopenia,chronic hemolysis, proteinuria and biliary stones	[22]
21	Chinese	exon 6	c.581G > T	p. Gly194Val	Missense	Hemolytic anemia, thrombocytopenia,ecchymosis, petechiae, decreased liver function, jaundice and fever	Department of Hematology,303rd Hospital of the People's Liberation Army (Aug 15, 2018)
		exon 18	c.2209T > C	p. Cys737Arg	Missense		
22	Not available	exon 23	c.2863dup	p. Trp955fs	Frameshift	Not available	Mendelics (May 28, 2019)
23	Not available	_	c.3044 + 1G > A	_	Splice site	Not available	GeneDx (Jan 29, 2019)
24	Chinese	exon 11	c.1335delC	p. Phe445LeufsTer52	Frameshift	Plastocytopenia, neurological abnormalities and renal involvement; Had a chronic relapsing course, chronic hemolysis responded to plasma infusion	This study
		exon 18	c.2130C > G	p. Cys710Trp	Missense		

## Case presentation

Our patient was a 10-year-old female, who presented with discontinuous thrombocytopenia of 8 years, abnormal renal function of more than 2 years, cough of more than 10 days, and weakness of the left limb of 3 days. She was delivered at full term by caesarean section and weighed 3 kg, without asphyxia. Her parents were non-related and her mother was healthy during pregnancy. She was fed formula milk after birth. No family history of the genetic disease was available for assessment. She had no history of infectious diseases, no history of exposure to toxic substances or contaminated water, and no history of surgery, traumatic injury, blood transfusion, and drug or food allergies. Her vaccinations were performed as planned. At age of three, she was hospitalized for skin purpura, but no abnormality was observed in the bone marrow smear and she was diagnosed with “idiopathic thrombocytopenic purpura”. Afterward, her platelet count decreased after each cold, and she was hospitalized intermittently. At age of eight, the patient was admitted to our department for "thrombocytopenia". Her blood urea concentration was 7.78 mmol/L and total bilirubin was 41.6 μmol/L. After symptomatic treatment, her platelet count returned to a normal level, but the blood urea concentration was still elevated. The patient took prednisone for approximately 3 months. After discharge, she did not experience nausea, vomiting, swelling, reduced urine production, etc. At age of nine, the patient was hospitalized again in our department for "acute gastroenteritis". Her blood urea concentration was 8.86 mmol/L and total bilirubin was 65.2 μmol/L. After symptomatic treatment, the symptoms disappeared and she was discharged from the hospital. The patient did not attend a follow-up visit. She had experienced coughs 10 days before this admission, but no fever. The symptoms were not improved by taking oral cough syrup. In the last 3 days, she exhibited weakness in the left limb, but no fever or convulsions.

Physical examination: At the time of this admission when the patient was 10 years old, her body temperature was 36.9 °C, her pulse rate was 88/min, her breathing rate was 20/min, her blood pressure was 141/81 mmHg, and her weight was 46 kg. Her development was normal and consciousness was clear, but her gait was unstable. Slight pharyngeal hyperemia was observed, and bilateral tonsils were swollen in degree I. The breath in her lungs sounded thick, without dry or moist rales. No deformities of limbs and spine were observed. The muscle tension and volume of the limbs were normal. An ecchymosis was detected on the left knee and calf. The muscle strength of the right limb was normal. The muscle strength of the left upper limb was V-. The muscle strength of the left lower limb was normal. There was tenderness in the left gastrocnemius muscle, no sensory disturbances or pain, and no warmth disturbances. The rest of the respiratory, cardiovascular were normal.

Laboratory examination: The laboratory examination results of this admission showed anemia, thrombocytopenia, reticulocytosis, and hypertriglyceridemia. The results of her coagulation function analysis and renal function analysis also showed abnormalities. Her blood lactate dehydrogenase was elevated. Urinalysis revealed the presence of mild proteinuria. Meanwhile, her ADAMTS13 activity was lower than 2.5%. Other hepatic analyses, cerebrospinal fluid analyses, myocardial enzymes, and immunoassay were normal (Table 2).

**Table 2:** Laboratory investigation findings of the patient

Laboratory examination	Patient	Reference range
*Hematology and coagulation assays*		
White blood cell (× 10^9^/L)	6.37	8–10
Neutrophils (%)	70	50–70
Lymphocytes (%)	24.9	20–40
**Hemoglobin (g/L)**	***92***	120–140
**Hematocrit (%)**	***28.2***	35–45
**Reticulocyte (%)**	***3.09***	0.5–1.5
**Platelets (× 10**^**9**^** /L)**	***76***	100–300
Hypersensitive C-reactive protein (mg/L)	< 0.5	< 0.5
Erythrocyte Sedimentation Rate (mm/h)	18	0–20
**D-dimer (mg/L)**	***0,58***	< 0.3
Prothrombin activity (%)	***59.5***	75–100
Prothrombin time (sec)	***14.1***	12–14
Blood triglyceride (mmol/L)	***6.01***	< 1.13
*Immunoassay*		
Immunoglobulin A (g/L)	1.44	0.29–2.7
Immunoglobulin E (U/L)	21	< 100
Immunoglobulin G (g/L)	8.61	7–16.5
Immunoglobulin M (g/L)	1	0.5–2.6
Serum complement3 (g/L)	0.99	0.9–1.8
Serum complement4 (g/L)	0.15	0.1–0.4
Coombs test	Negative	Negative
**ADAMTS13 activity (%)**	**< *2.5***	68–130
*Hepatic analysis*		
Total bilirubin (μmol/L)	14.4	3.4–17.1
Direct bilirubin (μmol/L)	3.8	0–6.8
Alanine aminotransferase (U/L)	13	7–40
Aspartate transaminase (U/L)	26	13–35
*Renal analysis*		
**Urea (mmol/L)**	***14.41***	2.6–8.8
**Creatinine (μmol/L)**	***184***	41–81
**Uric acid (μmol/L)**	***596***	119–327
*Urinalysis*		
Qualitative protein analysis	**+**	Negative
*Cerebrospinal fluid analysis*		
Total cerebrospinal fluid protein (mg/L)	245	200–400
**Cerebrospinal Fluid Chloride (mmol/L)**	***130***	117–127
Cerebrospinal fluid glucose (mmol/L)	3.22	2.8–4.5
*Myocardial enzymes*		
Creatine Kinase (U/L)	162	25–170
**Lactate dehydrogenase (U/l)**	***531***	150–450
Cardiac troponin I (pg/mL)	30.49	< 200

Bold represents the patient had an abnormality in this indicator

**Fig. 1:**
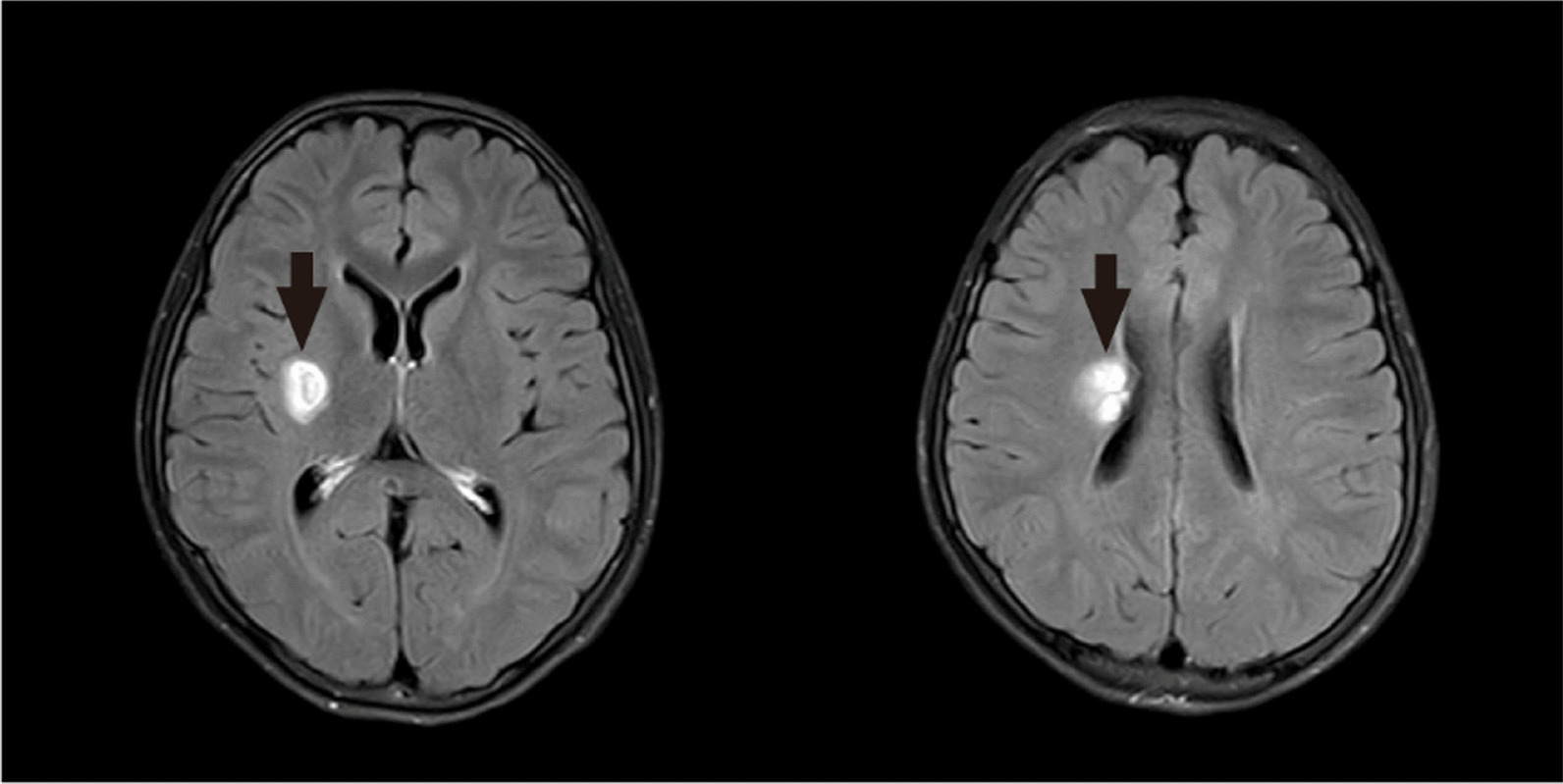
Cranial magnetic resonance images obtained from the patient. The black arrow shows the right side of the basal ganglia region-lateral ventricle lesion

Cranial magnetic resonance imaging: The right side of the basal ganglia region-lateral ventricle showed a flaky and slightly longer signal on T1 and T2 images, a short signal on T2 images of the lesion center, slight hyperintensity in the center of pressure water sequence lesions surrounded by a ring of hyperintensity, hyperintensity in most of the DWI sequence lesions with flaky hypointensity inside the lesions, hypointensity in the lesions corresponding to the ADC figure, an obviously enhanced signal in the lesion center on the enhanced scan and no obvious abnormal signals and non-enhanced lesions in the brain parenchyma (Fig. 1).

**Fig. 2:**
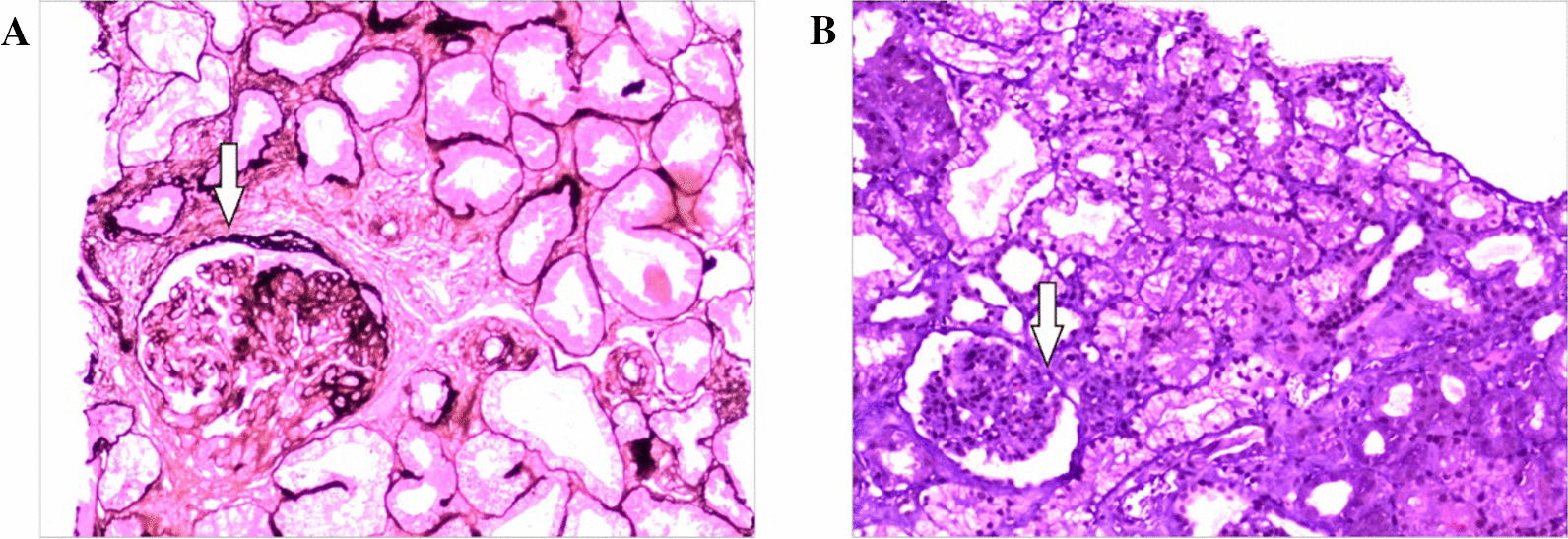
Images of the patient's renal needle biopsy. **A** The white arrow shows a crescent forming in the glomerulus (PASM). **B** The white arrow shows glomerular capillaries exhibiting glassy transformation and sclerosis (HE)

Renal needle biopsy: The images showed 1 renal cortex tissue, 11 glomeruli, 1 fibrocystic crescent, 1 cellular crescent, 1 region displaying focal segmental sclerosis, mild hyperplasia in the glomerular mesangium, the incomplete opening of the capillary loops, tubular atrophy, interstitial fibrosis, and low levels of inflammatory cell infiltration, vacuolar degeneration in renal tubule epithelial cells, calcification in certain tubule epithelial cells, a few swollen endothelial cells in the afferent vessel and the formation of a pink thrombus in the lumen of some small vessels. We considered that the girl may have suffered from nephropathy associated with the thrombotic microvasculature (Fig. 2). Immunofluorescence showed that only one glomerulus was found in the puncture specimen, C3 (++), C1q (±), IgM (++), IgA (±), and were deposited in the segment-mesangial zone, IgG (−).

**Fig. 3:**
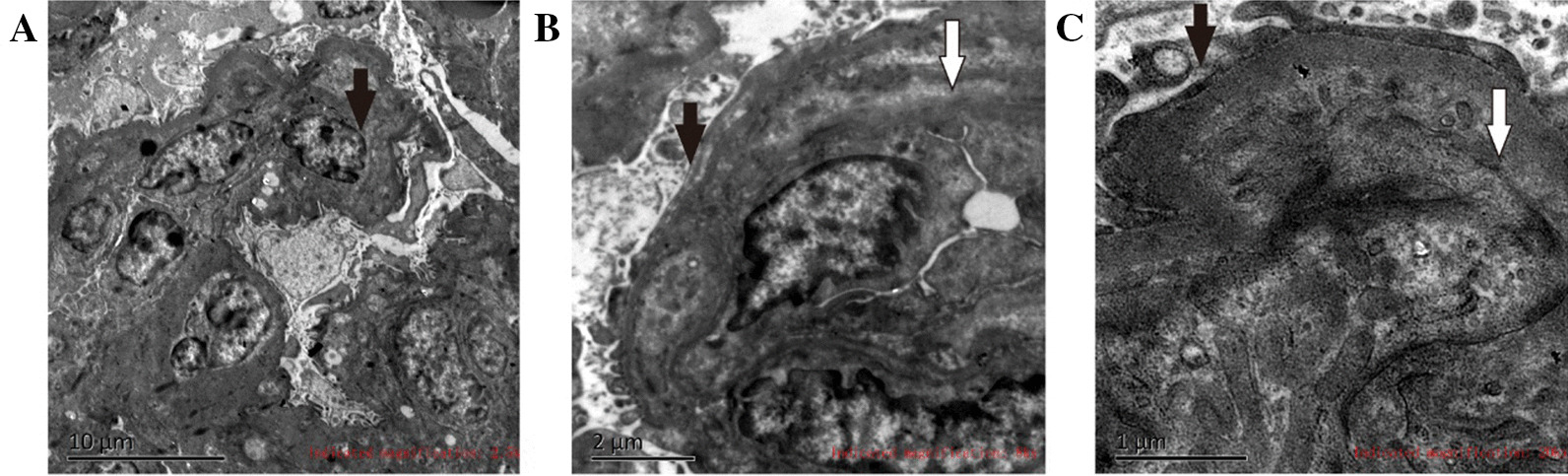
Electron microscopy images of the patient's renal needle biopsy. **A** The black arrow shows renal tubular epithelial cell edema and interstitial fibroproliferation. **B** The black arrow shows extensive podocyte fusion, and the white arrow shows thickening of the capillary basement membrane and endothelial loosening. **C** The black arrow shows the disappearance of epithelial podocytes from the visceral layer, and the white arrow shows necrotic endothelial cells and the deposition of particles

Electron microscopy images showed glomerular mesangial cell hyperplasia (1–3 cells/zone) with moderate nodular hyperplasia in the matrix, no obvious electron deposition, endothelial cell hyperplasia in part of the capillary (1–3 cells/vessel), cytoplasmic swelling, capillary basement membrane thickening (600–1000 nm), endothelial loosening, extensive podocyte fusion, edema in some renal tubular epithelial cells, interstitial fibroproliferation, two small arteries with necrotic endothelial cells, and the deposition of particles. The glomeruli showed moderate mesangial hyperplasia and basement membrane thickening and loosening, as well as hyperplasia and edema in endothelial cells (Fig. 3, Additional file 1: Fig. S1).

**Fig. 4:**
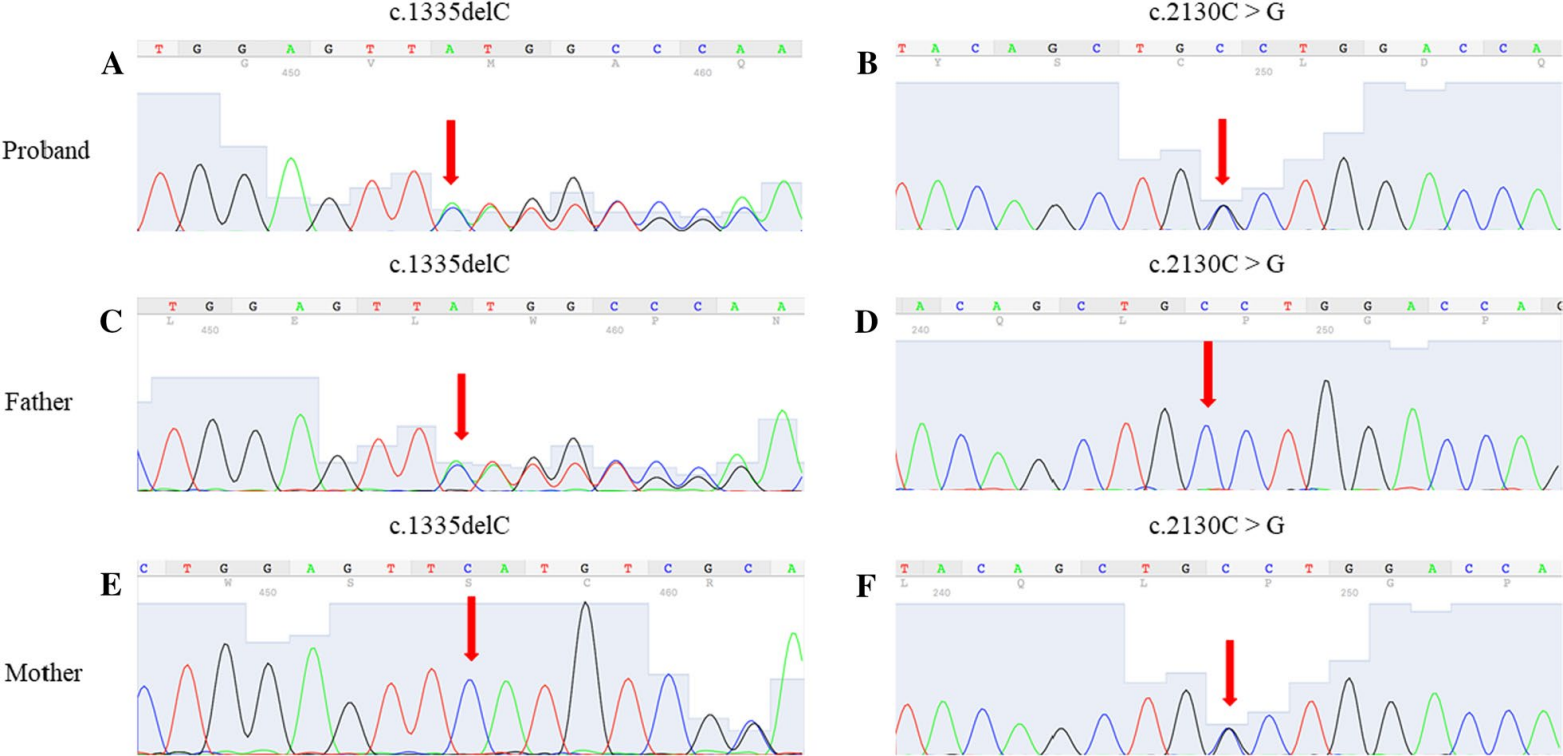
Sequences of *ADAMTS13* gene. **A** The proband carried the heterozygous mutation c.1335delC (p. Phe445LeufsTer52). **B** The proband carried the heterozygous mutation c.2130C > G (p. Cys710Trp). **C** The father of the proband carried the heterozygous mutation c.1335delC (p. Phe445LeufsTer52). **D** The father of the proband did not carry the mutation in site c.2130. **E** The mother of the proband did not carry the mutation in site c.1335. **F** The mother of the proband carried the heterozygous mutation c.2130C > G (p. Cys710Trp)

**Fig. 5:**
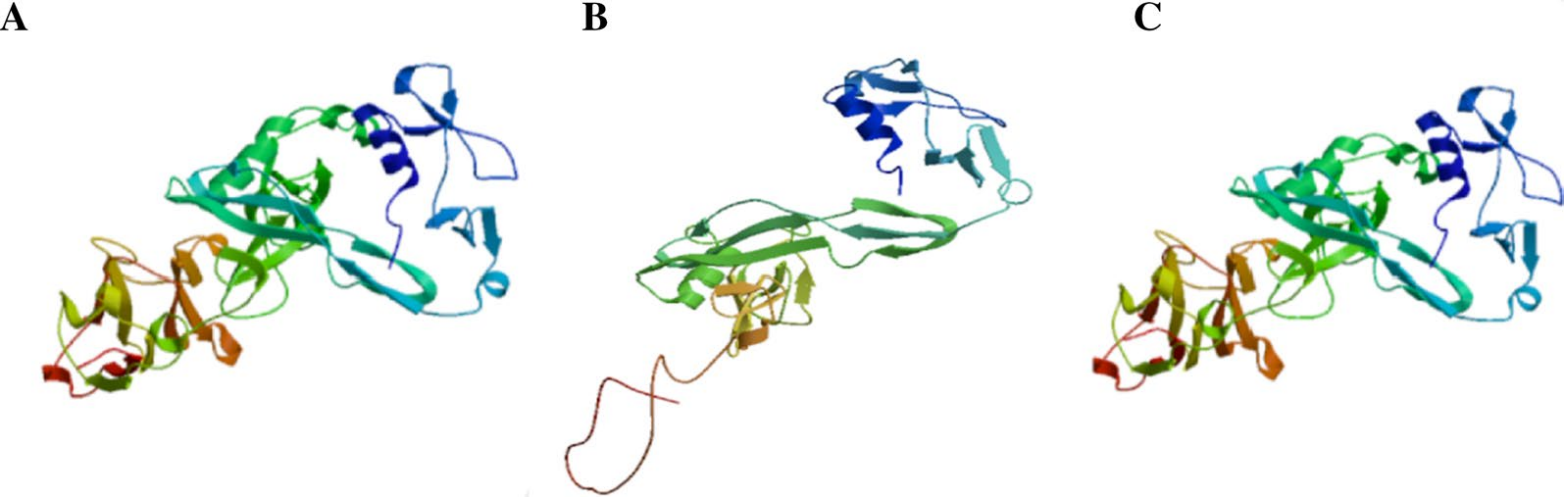
Structures of the protein encoded by the *ADAMTS13* gene. **A** Structure of the wild type ADAMTS13 protein. **B** Structure of the ADAMTS13 protein with the mutation in c.1335. **C** Structure of the ADAMTS13 protein with the mutation in c.2130

Gene sequencing: The patient’s *ADAMTS13* gene contains compound heterozygous nucleotide variations: c.1335delC (p. Phe445LeufsTer52) is a frameshift variation inherited from her father. c.2130C > G (p. Cys710Trp) is a missense variation inherited from her mother (Fig. 4). The frameshift variation changes the amino acid synthesis starting from phenylalanine 445 and ends at the 52nd amino acid after the change, resulting in a significant change in the structure of the protein. The missense variation causes amino acid 710 to be changed from Cysteine to Tryptophan (Fig. 5). We submitted these two variants to ClinVar, the submission numbers are SUB10260642 and SUB10296888 respectively.

Variant's pathogenicity: We qualified the functional impact of the reported variants by using in-silico algorithms. The pathogenicity of p. Cys710Trp was quantified by using Polymorphism Phenotyping v2 (PolyPhen2) and Protein Variation Effect Analyzer (PROVEAN) algorithms; since frameshift mutations are not applicable to the " PolyPhen2" and " PROVEAN " algorithms, we used "AutoPVS1" algorithm to quantify the pathogenicity of p. Phe445Leufs*52.

"PolyPhen2" algorithm shows that the pathogenicity of p. Cys710Trp mutation is predicted to be "PROBABLY DAMAGING" with a score of 1.000 (sensitivity: 0.00; specificity: 1.00). "PROVEAN " algorithm shows that the pathogenicity of p. Cys710Trp mutation is predicted to be "Deleterious" with a PROVEAN score of − 9.836 (cutoff = − 2.5). AutoPVS1 shows that the pathogenicity of p. Phe445Leufs*52 is "PSV1', which means very strong evidence of pathogenicity.

The girl was diagnosed with hereditary TTP, which was treated with oral nifedipine, dipyridamole, and coated aldehyde oxystarch capsules. She was discharged from the hospital after two weeks and continued to take the medications at home. In January 2018, the patient was readmitted to PICU in our hospital for the same disease. Her condition improved after treatment with plasma exchange, after which she was discharged.

## Discussion and conclusions

In this study, we analyzed the activity of patients' ADAMTS13 and performed genetic sequencing to diagnose the patient with USS. To the best of our knowledge, our report is the first report of mutations in these two pathogenic variants and is the first review that combines all the pathogenic mutations with available details.

Thrombotic thrombocytopenic purpura (TTP), which was first reported by Moschcowitz in 1924, is a rare kind of thrombotic microangiopathy (TMA). The incidence of TTP is 1–2/1,000,000. The male-to-female ratio is 2:3 [1]. Its main clinical manifestations include hemolytic anemia, severe thrombocytopenia, neurological abnormalities, fever, and renal involvement, which are called the “Penta sign”, and the first three manifestations are called the "Triad sign". The central nervous system and kidney are the two most common organs or systems affected by TTP [2]. TTP is characterized by a rapid onset, with early mortality of up to 90%, and some patients eventually progress to end-stage renal disease (ESRD) [3].

Based on our literature review of 29 *ADAMTS13* pathogenic mutations detected in 24 patients, we found that missense mutations (55%) are the most common type of mutation, followed by frameshift mutations (28%). Exons contained more mutations than introns. 11 patients (45.8%) showed the course of chronic relapsing TTP. 13 patients (54.2%) responded to plasma infusion. Nine patients (37.5%) had a history of hemolysis. (Table 1). Similar to half of the reported case, our patient also showed a chronic onset course and response to plasma exchange. Although neurological symptoms are a major symptom of TTP, it is rare in case reports. However, our patient has obvious neurological symptoms. Another outstanding feature is that there was no manifestation of fever or severe hemolytic anemia during this hospitalization, although her total bilirubin did increase significantly during her three hospitalizations between 8 and 9 years old. And after that she developed abnormal renal function. Jaundice is a common feature among the pediatric cases of USS, but the signs and symptoms of USS are not static. The Penta sign of USS is present in only 10% of patients [4]. This time she was admitted to the hospital due to cough and left limb weakness. Her hemolytic symptoms were chronic and did not occur acutely recently. This may be the reason why the patient had no jaundice with normal liver function this time.

The main pathogenic mechanism of TTP is related to the lack of the activity of the vWF-cleaving protease ADAMTS13, the abnormal release of vWF from vascular endothelial cells, and abnormal platelet activation, etc. ADAMTS13 contains disintegrin and metalloprotease domains with thrombospondins1 repeats and is the 13th member of the ADAMTS family. The gene is located on chromosome 9q34 and comprises 29 exons. ADAMTS13 was discovered in 1996, and its deficiency is unique to TTP and is not detected in other types of microvascular diseases, such as hemolysis and thrombosis. ADAMTS13 is synthesized by the liver and its main function is to cleave vWF from the vascular endothelial cell surface, in the blood circulation, and at vascular injury sites [5]. vWF is a type of macromolecular adhesive glycoprotein that is present as a polymer with a molecular weight of 500–20,000 in plasma and is an essential component of the normal hemostasis process [6]. Under physiological conditions, vWF is hydrolyzed by proteolytic enzymes to different degrees, among which ADAMTS13 is an important hydrolytic enzyme. *ADAMTS13* gene mutations or related IgG antibody generation result in a lack of activity of ADAMTS13 enzymes, the reduced clearance of vWF from vascular endothelial cells, the generation of the unusually large von-Willebrand-Factor (ULvWF), vWF and platelet aggregation into microvascular thrombosis, platelet consumption, microvascular thrombosis, and microangiopathy hemolytic anemia, causing tissue thromboembolism, failure and eventual death [7]. According to recent studies, the reduced activity of tissue plasminogen activator (tPA) is also associated with the occurrence of TTP. tPA converts fibrinogen into fibrinase, which degrades the vWF polymer in various sites and also cleaves adhesion molecules between the walls of blood vessels and the platelet. Therefore, the reduction of the activity of tPA results in similar effects to decreased ADAMTS13 activity, resulting in the formation of microvascular thrombosis and producing a series of pathological changes, suggesting that a reduction in the tPA activity is one of the most likely causes of idiopathic TTP [8]. Unfortunately, a tPA test was not performed on this patient.

The frameshift mutation c.1335delC is located in the cysteine-rich domain, and all other TSP1 repeat domains after the appearance of the stop codon disappear. This may be related to the early onset of the disease and the repeated chronic course phenotype. The missense mutation c.2130C > G is located between the spacer domain and the TSP1 domain, which may indicate a lighter TTP phenotype [9]. We speculate that in this compound heterozygous mutation, the frameshift mutation is the main cause of severe symptoms in patients. However, more information about the relationship between the genotype and phenotype of these two mutations requires the support of in vitro experiments.

TTP is classified into hereditary and acquired forms, the latter of which is classified as idiopathic and secondary, according to the presence or absence of the primary disease. Hereditary TTP, also known as Upshaw–Schulman syndrome (USS), is a rare autosomal recessive hereditary disease caused by a decrease in or lack of enzyme activity due to a homozygous or heterozygous mutation of the *ADAMTS13* gene [10]. USS has manifested as two phenotypes: an early-onset phenotype that has been observed in newborns and the delayed-onset phenotype that usually does not produce symptoms in childhood, whereas the first symptoms occur after puberty or in adulthood [11]. The patient in this case study experienced symptoms at the age of 2 and thus was diagnosed with early-onset USS. Idiopathic TTP is the main clinical type of acquired TTP. Autoantibodies or inhibitors of ADAMTS13 in patients, mainly the IgG type, results in a decrease in or lack of ADAMTS13 enzyme activity, which usually occurs in patients with genetic risk factors, more than 95% of whom are adults [12]. Secondary TTP is caused by an infection, drugs, tumors, autoimmune diseases, hematopoietic stem cell transplantation, and other factors; this type of TTP is characterized by complicated pathogenesis and poor prognosis. Regardless of whether the patient is diagnosed with hereditary or acquired TTP, plasma levels of ADAMTS13 enzymes must be detected before the administration of plasma treatment. For patients with enzyme activity < 10%, ADAMTS13 antibodies should be detected. If the antibody test is negative, sequencing should be performed to identify mutations in exons of the *ADAMTS13* gene for the differential diagnosis of hereditary TTP in the patient [13]. If the antibody test is positive, the patient should be diagnosed with secondary TTP. The detection of ADAMTS13 activity and antibodies is not merely conducive to a differential diagnosis but also the application of more targeted treatments, predictions of relapse, and the determination of long-term prognoses of patients with TTP [5]. In this case, the relevant antibodies were not detected.

Currently, the diagnosis of TTP requires the presence of the following features: the clinical manifestations of TTP; typical changes in blood cell counts and biochemical parameters; a significant decrease in the plasma level of ADAMTS13 activity, as the ADAMTS13 inhibitor is often detected in patients with idiopathic TTP, but cannot be detected in some patients and patients with the hemolytic uremic syndrome (HUS), disseminated intravascular coagulation (DIC), hemolysis, elevated liver enzymes, and thrombocytopenia syndrome (HELLP), but not Evans syndrome and eclampsia. The disease is dangerous and has a high fatality rate. Aggressive treatment should be initiated as soon as possible after the patient receives a definitive diagnosis or is highly suspected of having a slight or severe case of the disease. Plasma replacement therapy is preferred, followed by fresh (frozen) plasma infusion and medication. In highly suspected and confirmed cases, platelet transfusion should be cautiously administered and should be considered for use only when severe life-threatening bleeding occurs. The main treatment for acute onset hereditary TTP is plasma exchange/plasma infusion. The main treatment for secondary TTP is plasma exchange, and if necessary, it can be combined with immunosuppressive agents [14]. Approximately 30–40% of patients with TTP may experience a relapse within 10 years of complete remission. In this situation, plasma exchange therapy should be resumed as soon as possible [15]. The patient in our case study exhibited a stable condition after plasma exchange and was discharged from the hospital.

In this case report, we described a 10-year-old girl with two compound heterozygous *ADAMTS13* variants. The patient was diagnosed with hereditary TTP. She showed neurological symptoms without serious coagulation abnormalities. The relationship between genotype and phenotype in these two mutations requires further in vitro investigation.

## Supplementary Information

**Additional file 1.** Electron microscopy images of the patient's renal needle biopsy.

## Data Availability

The datasets generated and analyzed during the current study are available in the ClinVar archive, under the Accession Number SCV001815641 and SCV001810646. The below listed databases are used for data analysis. ClinVar: https://www.ncbi.nlm.nih.gov/clinvar/; PolyPhen2: http://genetics.bwh.harvard.edu/pph2/; PROVEAN: http://provean.jcvi.org/; AutoPVS1: http://autopvs1.genetics.bgi.com/.

## Abbreviations

TTP: thrombotic thrombocytopenic purpura
USS: Upshaw–Schulman syndrome
TMA: thrombotic microangiopathy
ESRD: end-stage renal disease
vWF: Von Willebrand factor
ULvWF: unusually large von-Willebrand-Factor
tPA: tissue plasminogen activator
HUS: hemolytic uremic syndrome
DIC: disseminated intravascular coagulation
