# Supplementary material for: An ADAMTS13 mutation that causes hereditary thrombotic thrombocytopenic purpura: a case report and literature review
Source: BMC Med Genomics. 2021 Oct 26;14:252. doi: 10.1186/s12920-021-01099-3 (PMC8549186; doi:10.1186/s12920-021-01099-3)
Supplement: Supplementary file 1 — Additional file 1. Electron microscopy images of the patient's renal needle biopsy. [file 12920_2021_1099_MOESM1_ESM.docx]

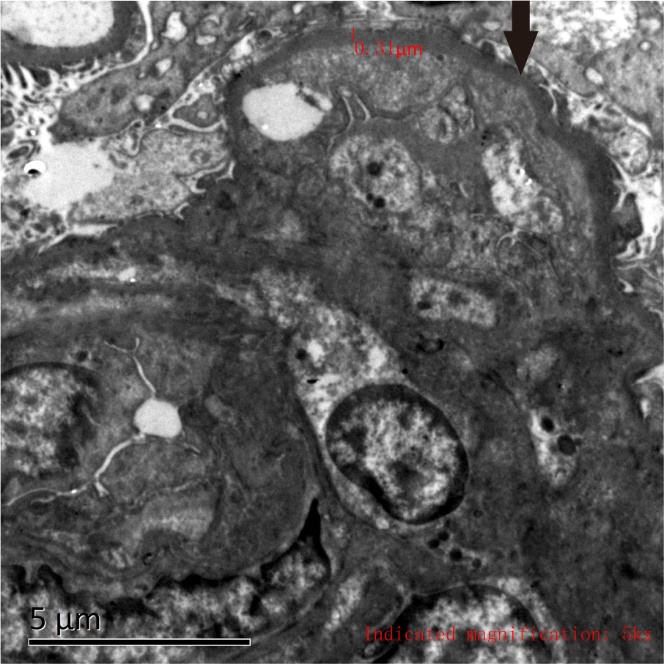


**Additional file 1: Figure S1**: Electron microscopy images of the patient's renal needle biopsy. The black arrow shows basement membrane thickening and podocyte fusion.
